# Immaterial vs. Material

**Published:** 1885-04

**Authors:** R. B. Johnstone

**Affiliations:** Pittsford, N. Y.


### IMMATERIAL vs. MATERIAL.

Dr. R. B. Johnstone, Pittsford, N. Y.

"Tyndall says that the whole mass of particles which give the blue to the sky could be packed together in a lady's toilet-box."—*New England Medical Gazette, January,* 1881.

C. Wesselhoeft, M. D., Boston, Mass., in the *New England Medical Gazette*, 1880, in an article intending to show the limit to the divisibilty of matter, especially relating to the preparation of homœopathic potencies, says in relation to gold, as an example, that one grain of gold contains 46,080,000,000 particles of gold reduced to its utmost limit of $\frac{1}{2000}$ of a millimeter in diameter; that while the first cent. trit. would contain the whole 46,080,000,000 particles, the sixth trituration would contain but $4\frac{3}{5}$ particles, each $\frac{1}{2000}$ of a millimeter, thus showing, according to his figures, that of one hundred powders of one grain, each of the sixth cent. trituration, ninety-five of them would be non-medicinal.

Now, C. Wesselhoeft, M. D., may have an excellent microscope, and know how to handle it in the highest Boston style. He tells us that he has measured the smallest particle which he saw, and that its diameter was a trifle more than $\frac{1}{2000}$ of a millimeter. It is to be presumed that in his investigations he made use of the highest power instrument. There is one thing, however, which he neglected to do, *i. e.*, to give us the diameter of the smallest particle of gold which his instrument did not reveal, and which he consequently did not see. Another oversight in his investigations: He neglects to note for our benefit the molecular or life motion with which all matter is endowed, both organic and inorganic. He fails to note these important facts. His argument as to the divisibility of matter as demonstrated by his microscope is as conclusive as the assertion that no object less than seventy feet in length exists upon the surface of the moon, because the telescope fails to reveal smaller objects, notwithstanding the fact that thousands of physicians have demonstrated tens of thousands of times that remarkable cures have been wrought by potencies far above the sixth. However, for the sake of argument, we will concede that matter is extinct after the tenth centesimal. Even conceding this, it does not injure our position. Nevertheless, we do not believe that medical matter is entirely absent in the thirtieth or two hundredth, even though the microscope may not be able to show its presence, and, furthermore, we do not believe the presence of matter is essential to the prompt curative action of a remedy in a true homœopathic sense. We believe that the toxical and curative effect of drugs, crude or potentized, is not due to the material tangible to our senses, but rather to the force developed by the peculiar molecular motion, dynamic or life force, which is peculiar to each individual drug, whereby it receives its individuality. In the crude drug we have the toxical effects predominating over the finer shades of molecular or dynamic action. The process of potentization destroys the more violent action, while it liberates the truly curative dynamic force, or molecular vibrations. Each remedy thus potentized maintains its individuality of action from the lowest to the highest potency; it always remains the same, differing only in the intensity and depth of action. Whatever the potency may be, its symptoms, exhibited through the mind or body, retain its stamp of individuality. They have their affinity for different portions of the body, their action are known as recorded in the provings, and according to their known effects and affinities are they prescribed.

I have never met a man who has seen *disease*, even aided by a microscope, but all acknowledge to have seen the results of disease without the aid of a glass. In other words, we can all see the effects of the immaterial cause upon the materials. We see its result, and call that result disease. We believe that disease is not the result of action of material within our tissues, but rather the immaterial force which changes the normal rates of vibrations of nerve cells, which control the properties of nutrition and power of motion. The cells may be located at the origin, within the trunk, or at the periphery of the nerve. Whatever their position within the living body, their foundation structure is practically the same, differing only in their normal rates of vibration. Anything which may disturb this normal vibratory motion gives origin to a train of symptoms subjective and objective, which are disease. A normal rate of molecular vibrations in one portion of the body if transferred to another portion would be abnormal, and produce a disturbance recognized by the symptoms thereof as disease. The deposit of calcareous matter in bony tissue is the result of a normal nutrition controlled by the nerves acting under a healthy or normal rate of molecular vibrations. Should this normal rate be disturbed, the calcareous matter will be supplied in too large or too small quantities. Or should the normal rate controlling calcareous deposit be transferred to those nerves which control the growth of the skin, we have a diseased condition resulting in calcareous matter being deposited in abundant quantities within the skin. Now to remove this deposit by mechanical or chemical means will not cure the difficulty, because the trouble lies at the fountain head of life, or among the molecules which originate the impulse or vibration or dynamic power which controls the nutritive function of those parts in which the objective disease exists. Now, should we cut down upon these controlling nerves, examine its entire length, we would discover nothing, because our microscopes are not capable of discerning this dynamic molecular vibration or life power. Yet no one dare say that this calcareous deposit is not due to a nutrition under the direct control of nerves whose function it is to properly deposit the right kind of nourishment in the right place. Yet should it deposit calcareous matter within the heart it is disease.

Notwithstanding this terrible havoc and destruction by malnutrition depended upon a cause which is undiscoverable by our most powerful microscopes, physicians continue to rail and beat the air madly because we succeed in curing disease with dynamic power, as exhibited in high potencies deprived of material incumbrance and endowed with the molecular vibrations peculiar to the material from which the remedy is derived; we do it in less time, in a safer manner, than those who use crude drugs, no drugs, or low potencies; and by clearly intelligible reasons, Dr. Gregg, of Buffalo, for fifteen years has met with uniform success in the treatment of diphtheria with high potencies. In my own experience I have been equally successful since adopting this strict homœopathic method, treating upward of one hundred cases of true diphtheria, averaging not over one week's illness. High potencies cure diphtheria by dynamic power because they control the abnormal rates of vibration, which is the cause of the disease, by reason of similarity of action. Is this discouraging? Does it indicate the non-medicinal properties of high potencies? I do not deny the same might have been done with low potencies or material drugs, but is it? and if so, has the success been any greater? Does it not rather indicate that the cure was not due to the material, but rather to the immaterial dynamic power inherent with the drug used. Is it not time that these materialistic investigations should cease? Has not the old school been making them for three thousand years, every few years discovering the causes of disease, this new theory to be abandoned for a new and more recent discovery, which is received for a short time, and in turn gives way to another recent discovery more wild in theory than those before it, each newly discovered cause accompanied by its specific, yet men die?

When will men begin to look for real causes, and not be willing to abide by the verdict of somebody's at best imperfect microscope? At this present time the whole world is idolizing Koch, of Berlin, because he has succeeded in showing us a result of cholera, palming it off upon the susceptible public as the cause. He, however, does not venture to name the remedy; he leaves that for Homœopathy to tell. He tells us that the germ is more active in wet than dry weather. Note the cholera reports and see how wonderfully the mortality falls after a rainstorm. If he is incorrect in this, may he not be in fault in all? The microbe may contain the potency of cholera, but is not the only thing that may contain it. Its absence alone does not indicate that a district may not, or will not, be invaded. The molecular construction of the atmosphere may be so altered from its normal rate of vibration, that it so acts upon the living organism as to change the normal vibration of controlling nerves, as exhibited in the illustration heretofore, depositing the right material in the wrong place, destroying the integrity of certain natural functions within the body, which gives rise to cholera.

The whole body is impregnated with the peculiar dynamic cause which perpetuates the disease, throwing off excretions containing this same dynamic cholera potency, which is capable of reproducing after its kind—that is, imparting to healthy tissue its abnormal rates of vibrations, which is cholera. Koch's investigations are in the right direction to bring his followers to us; why should we bow our knee to the opposition? Sooner or later they will recognize that they have been playing a game of checkers among themselves, and it matters little which way they jump, they land on the same block, of dynamic force, and are ours. Nearer and nearer each day they come to the truth, slow, by degrees, 'tis true, but the end is inevitable. Closer and closer to the wall we push them, gradually forced to acknowledge, one by one, the great truths promulgated by Hahnemann. Gradually they are absorbing the truth and utilizing it, while a lot of so-called homœopaths are begging to be permitted to use their cast-off, threadbare theories. They even acknowledge our Psora theory—see quotations in the *Organon*, Vol. III, page 212, also *N. E. Medical Gazette*, December, 1883, page 368.

We have attempted to show the true cause of disease. We see how by the changed molecular vibrations from the normal standard disease results. We have seen its immaterial force.

Thus it is with all medicines, each one endowed with a dynamic power, molecular motion, or life force peculiar to itself; having affinity for certain and fixed molecules within the living organism, capable of imparting to them the peculiar motion possessed by themselves, producing phenomena similar to that which is the cause of so-called disease.

The cure consists in sending to that part diseased a potency, dynamic power remedy having the same rate of vibration as that abnormal molecule which produces the morbid condition exhibited to our senses by the totality of symptoms. No two things can occupy the same place at once. The disease and medicine are neutralized, the cure begins and in due time is completed. Is anything more reasonable, more satisfactory, and so full of benefit to the human family? Does it not obliterate the disease in its entire extent, in the shortest, most reliable, and safest manner, according to clearly intelligible reasons? I do not deny the efficiency of low potencies, neither do I believe in the limit of the divisibility of matter. High or low, let it be the totality of symptoms, the smallest dose, the single remedy, and *Similia similibus curantur*.

